# Design of a Novel Microlens Array and Imaging System for Light Fields

**DOI:** 10.3390/mi15091166

**Published:** 2024-09-21

**Authors:** Yifeng Li, Pangyue Li, Xinyan Zheng, Huachen Liu, Yiran Zhao, Xueping Sun, Weiguo Liu, Shun Zhou

**Affiliations:** School of Optoelectronic Engineering, Xi’an Technological University, Xi’an 710021, China; 18829515777@163.com (Y.L.); lpy971230@163.com (P.L.); yanxinnm@163.com (X.Z.); 15389287986@163.com (H.L.); m13279606007@163.com (Y.Z.); sunxueping.1988@163.com (X.S.); wgliu@163.com (W.L.)

**Keywords:** light field imaging system, microlens array, multifocal length, optical design

## Abstract

Light field cameras are unsuitable for further acquisition of high-quality images due to their small depth of field, insufficient spatial resolution, and poor imaging quality. To address these issues, we proposed a novel four-focal-square microlens and light field system. A square aspheric microlens array with four orthogonal focal lengths was designed, in which the aperture of a single lens was 100 μm. The square arrangement improves pixel utilization, the four focal lengths increase the depth of field, and the aspheric improves image quality. The simulations demonstrate pixel utilization rates exceeding 90%, depth-of-field ranges 6.57 times that of a single focal length, and image quality is significantly improved. We have provided a potential solution for improving the depth of field and image quality of the light field imaging system.

## 1. Introduction

**Figure 1:**
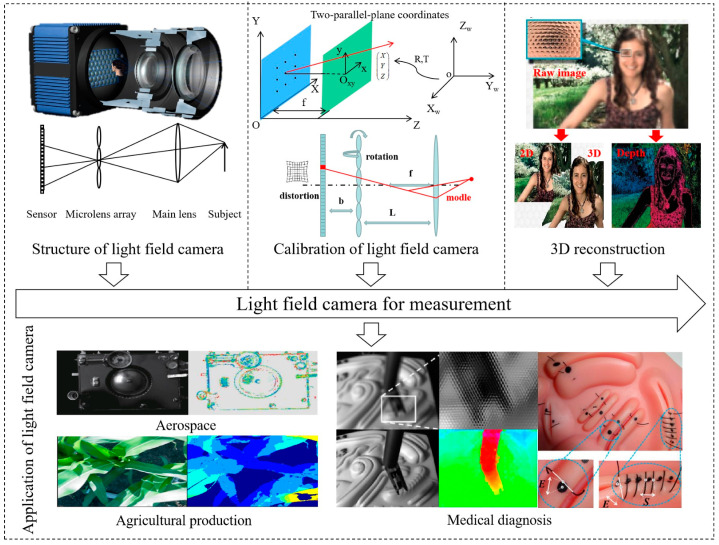
Technology of light field cameras and their application in industrial inspection. Reprinted from ref. [25].

As a new type of optical imaging technology, light field imaging [1,2,3,4,5] is a crucial player in the growing field of photoelectric detection. A light field imaging technology concept was first proposed in 1936 by Alexander Gershun [6]. With computer and digital camera technology developed since the 1990s the 20th century, light field imaging has achieved significant advances with refocusing capability [7,8], multi-view information [9,10,11], depth perception [12,13], compact structure, and fast imaging [14]. Numerous applications have been proposed for light field imaging, ranging from computer vision [15,16] and industrial inspection [17,18,19] to medical imaging [20,21] and autonomous driving [22]. The rise of light field cameras is especially evident in the field of industrial measurement, as shown in Figure 1. Depending on the hardware structures, there are two main types of light field imaging systems: light field cameras and camera arrays [23]. Light field camera uses a microlens array as their core elements and one sensor for capturing both light intensity and direction simultaneously so that they can realize the function of “taking pictures first and then focusing”. As a result, light field cameras can be used in precision inspection fields such as the inspection of workpieces in 3D, the inspection of chips for pins, and the detection of defects in screens of cellphones for delamination [24].

As a result of secondary imaging of the microlens array, the light field camera produces information about light intensity and direction simultaneously. In contrast to traditional optical imaging systems, this method sacrifices spatial resolution to obtain angular resolution. Research has been conducted on this problem by both domestic and foreign researchers, and various solutions have been proposed. To improve spatial resolution, Shanghai Jiao Tong University’s research team proposed a new light-scan-based light field framework (LSLF) [26]. The LSLF camera replaces the traditional square/hexagonal microlens array and area-scan COMS with the cylindrical microlens array and line-scan CMOS, resulting in significantly improving the spatial resolution of light field imaging at a price of slightly decreased depth of field (DOF) and depth resolution. YOUNG-GIL CHA (Korea Advanced Institute of Science and Technology) reported a microlens array camera with variable apertures for high dynamic range (HDR) imaging by using microlens arrays with various sizes of apertures [27]. This reconstructed HDR image possesses an extensive dynamic range and an image resolution equivalent to the maximum MTF50 value in an LDR image. Hyun Myung Kim (Gwangju Institute of Science and Technology) has developed a vari-focal light field camera [28]. Unlike traditional light field cameras, this camera has a vari-focal lens as its main lens, extending the DOF to 15 m. Remarkably, the above teams have achieved some results in extending the spatial resolution and depth of field of light field cameras. Despite this, it is still a challenging problem to achieve high-resolution, wide depth of field, and high-quality imaging with light field cameras. Consequently, this paper proposed a novel microlens array structure and light field imaging system to overcome this issue.

This paper describes a novel focused light field imaging system designed to enhance spatial resolution and depth of field. In the system, a self-designed four-focal-length microlens array functions as a secondary lens after the beam passes through the main lens, and then a detector detects and records the intensity and direction of the light. Due to the design of the micro square lens array, high spatial resolution, wide depth-of-field range, high imaging quality, and compact optical structure are achieved. The new light field imaging system designed in this paper has many advantages over comparable methods.

## 2. Principle of the New Light Field Imaging System

### 2.1. System Scenario

**Figure 2:**
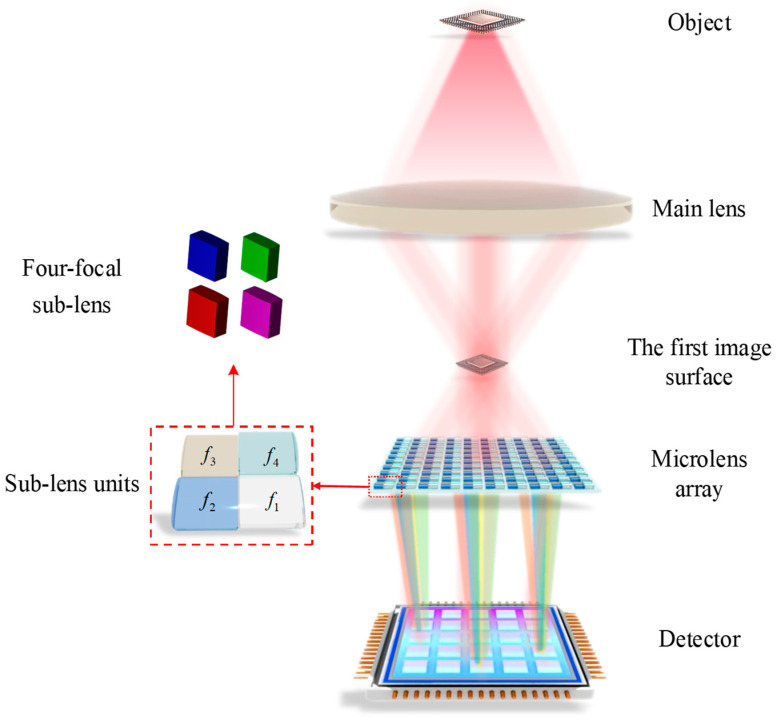
Schematic diagram of the new light field imaging system.

The imaging system designed in this paper is a focused light field imaging system, which overcomes the constraints of spatial resolution and angular resolution in traditional light field imaging systems. As illustrated in Figure 2, an overview of the new type of focused light field imaging system is presented. This system has three main components: a main lens, a novel microlens array, and a detector. A novel microlens array is located between the main lens and the sensor but not in the focal plane. In this design mode, the main lens can be re-imaged to improve the resolution of the imaging system.

This paper proposes a novel four-focal-length microlens array structure as the core device for optical field imaging. Due to its square arrangement, it can receive a higher amount of light energy, improving the detector’s pixel efficiency. When four focal lengths are designed, adjacent sub-microlens can be connected through the depth-of-field boundary, thereby expanding the depth-of-field range. To improve image quality and limit the effects of aberrations, each sub-microlens is designed to be aspherical. Thanks to this novel microlens array structure, the system is capable of obtaining high-resolution imaging, a large depth of field, and high-quality images.

### 2.2. Design of Imaging System Structure

In this paper, Keplerian types of focused light field imaging structures are studied in order to improve the spatial resolution of light field imaging.

**Figure 3:**
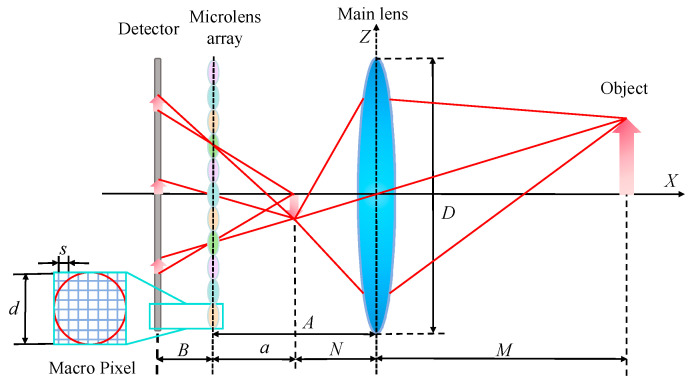
Optical path structure diagram of Keplerian light field imaging system.

Figure 3 illustrates the structure of a Keplerian-type light field imaging system. (To represent the imaging process, the individual optical elements have been magnified differently in the diagram.) In Figure 3, *M* represents the distance between the object and the main lens, *N* represents the distance between the image of the object and the main lens, *A* represents the distance between the main lens and the microlens array, and *B* represents the distance between the microlens array and the detector. After determining the object distance *M*, the Gaussian formula can be used to calculate the image distance *N* of the target after being imaged by the main lens. Additionally, *N* can calculate parameters *A* and *B*.

**Figure 4:**
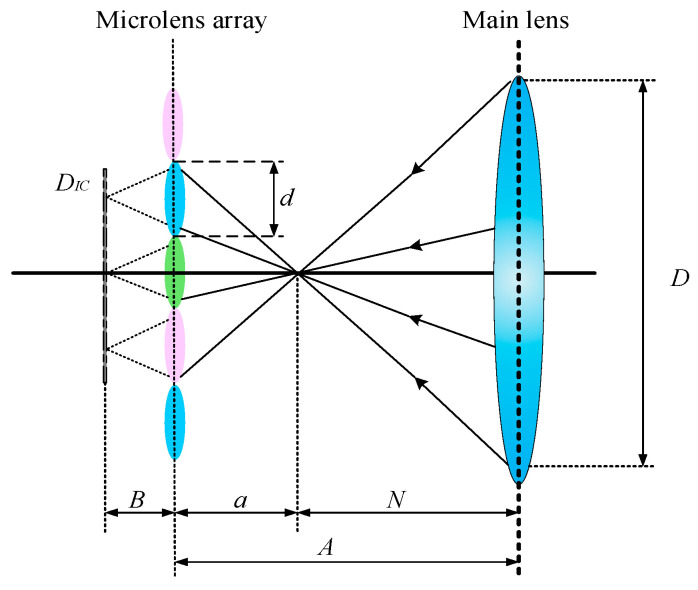
Schematic representation of the same object being imaged on multiple microlens.

To obtain depth information in light field imaging, multiple sub-microlens are required to image the target multiple times. After that, using the Gaussian formula, the object distance is calculated from the parallax information between each image point. Assuming that the object point is imaged by *n* sub-microlens, as shown in Figure 4 (*n* is 3), it can be obtained through the geometric relationship and imaging law:

(1) $$\frac{1}{a}+\frac{1}{B}=\frac{1}{f},$$

(2) $$\frac{a}{B}=\frac{n\cdot d}{D},$$

(3) A=a+N,

In Equations (1)–(3), *a* represents the object distance of the secondary imaging of the microlens array; *d* denotes the aperture of the sub-lens; *f* denotes the focal length of the sub-lens; and *D* indicates the aperture of the main lens. The simultaneous Equations (1)–(3) can be used to calculate *A* and *B* in the following way:

(4) $$A=\frac{\left(N+f\right)+\sqrt{N^{2}+f^{2}+\left(2+4n\right)Nf}}{2},$$

(5) $$B=\frac{\left(2n+1\right)Nf+f^{2}+\sqrt{N^{2}+f^{2}+\left(2+4n\right)Nf}}{2nN},$$

### 2.3. Design Method for a Novel Four-Focal-Length Square Microlens Array

#### 2.3.1. Analysis of Microlens Array Arrangement

**Figure 5:**
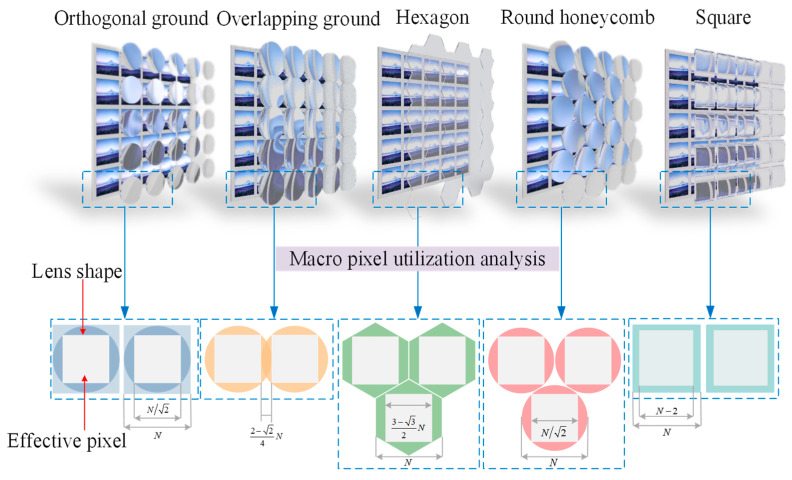
An illustration of the spatial arrangement of different microlens arrays and the effective pixel area.

The arrangement of the microlens array in a light field imaging system has an important impact on image quality, so it should be taken into account when designing the system. As far as light field imaging systems are concerned, microlens arrays are generally arranged in the following ways: round orthogonal arrangement, square arrangement, overlapping rows in circles, round honeycomb arrangement, and hexagonal arrangement. As illustrated in Figure 5, these arrangement methods have different spatial structures and effective pixel utilization areas.

In Figure 5, the round orthogonal arrangement is currently the most common arrangement of microlens arrays used in light field imaging systems. As a result, the image received by the detector after imaging is also tangentially arranged in circles. Due to this, pixels in macropixels will inevitably have areas that do not receive incident light, and these pixels will not be able to record light field information, thereby reducing the effective use of the sensor. Assuming that the diameter of the circular sub-microlens is equivalent to *n* pixel units, the pixel utilization of the sensor can be calculated by Equation (6). where R = *n* − 1, the number of pixels corresponding to the macropixel must be an integer, so the method of rounding down is used. By increasing the number of pixels *n* covered by the sub-microlens, the pixel utilization will increase and reach up to 50% in the round orthogonal arrangement.

(6) $$\eta =\frac{{\left[\sqrt{2}(n-1)\right]}^{2}}{{\left(2n\right)}^{2}}\approx \frac{{\left(n-1\right)}^{2}}{2n^{2}},$$

It is most efficient to increase the filling rate of the microlens array and adopt the aperture shape and arrangement with a high filling factor to improve pixel utilization. Several optimized microlens array arrangement methods can be used to increase pixel utilization, which include overlapping rows in circles, round honeycomb arrangement, hexagonal arrangement, and square arrangement. In Equations (7)–(10), their pixel utilization can be calculated.

(7) $$\eta =\frac{{\left[\frac{2+\sqrt{2}}{4}(n-1)\right]}^{2}}{n^{2}},$$

(8) $$\eta ={\left[\frac{\sqrt{2}}{2+\sqrt{2}-\sqrt{3}}\right]}^{2}\cdot \frac{{\left(n-1\right)}^{2}}{n^{2}},$$

(9) $$\eta =\frac{{\left[2\frac{\sqrt{3}}{\sqrt{3}+1}n\right]}^{2}}{\frac{3\sqrt{3}}{2}n^{2}},$$

(10) $$\eta =\frac{{\left(n-2\right)}^{2}}{n^{2}},$$

**Figure 6:**
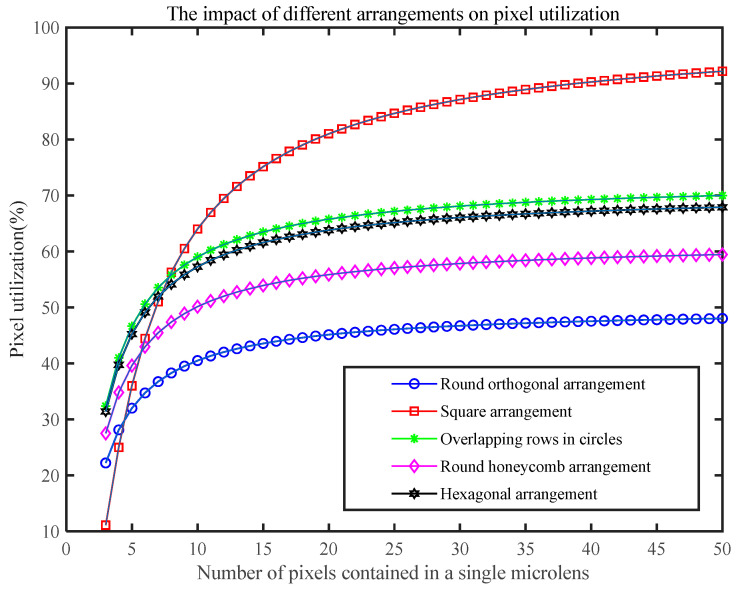
Comparison of pixel utilization of microlens arrays with different arrangement methods.

In Figure 6, we calculated the pixel utilization rate of round orthogonal arrangement, square arrangement, overlapping rows in circles, round honeycomb arrangement, and hexagonal arrangement by taking *n* from 3 to 50, respectively. As shown in the figure, the pixel utilization rate of the microlens array with five arrangement methods increases with increasing *n*. With *n* = 50, the square arrangement’s pixel utilization rate reaches 90%, which is significantly higher than the pixel utilization rate of other layout methods. Thus, a square arrangement is chosen in the design of this paper to ensure higher pixel utilization, and a large number of pixels are located in the sub-microlens.

#### 2.3.2. Theory of Multifocal Microlens Arrays to Extend the Depth of Field

In secondary imaging, the microlens array in the light field imaging system is imaged from multiple angles through multiple sub-microlens. The microlens array has a small depth-of-field range due to the relatively small aperture of each sub-microlens. Therefore, increasing this range is a problem worth exploring. In this study, a comparison is made between the depth of field of the traditional optical system and the light field imaging system, as shown in Equation (11):

(11) $$\rho =\frac{1}{\left(\frac{B}{f}-1\right)},B\neq f,$$

**Figure 7:**
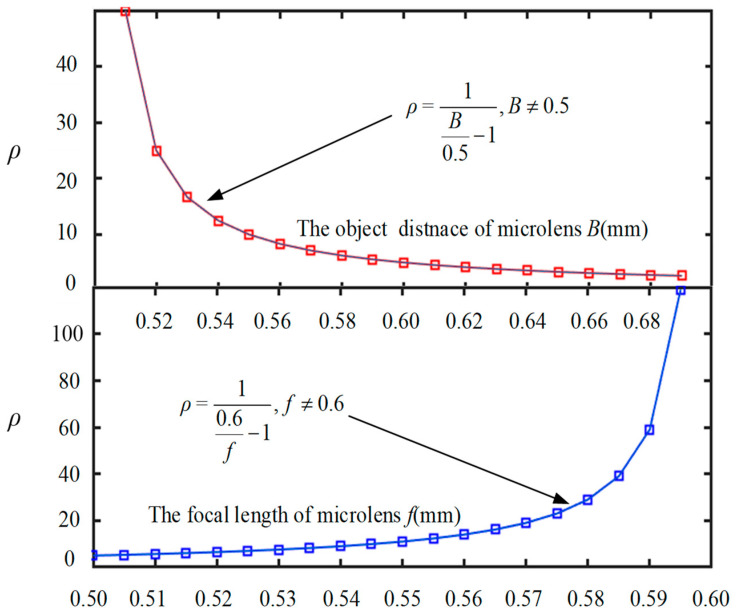
Effect of parameters *B* and *f* on depth of field.

In Equation (11), it can be seen that changing the proportion of parameters *B* and *f* extends the depth of field of the light field imaging system. Figure 7 depicts the effect of changing parameters *B* and *f*, respectively, on the depth of field of the entire light field imaging system. In a microlens array structure, increasing *f* or decreasing *B* can effectively enhance the depth of field. In view of the fact that *B* is a system parameter and it is closely related to the design of the system structure, it is difficult to change; therefore, it is best to increase *f* of a single sub-microlens to increase the depth of field. However, in terms of increasing focal length, there are limits to the depth of field that can be achieved.

**Figure 8:**
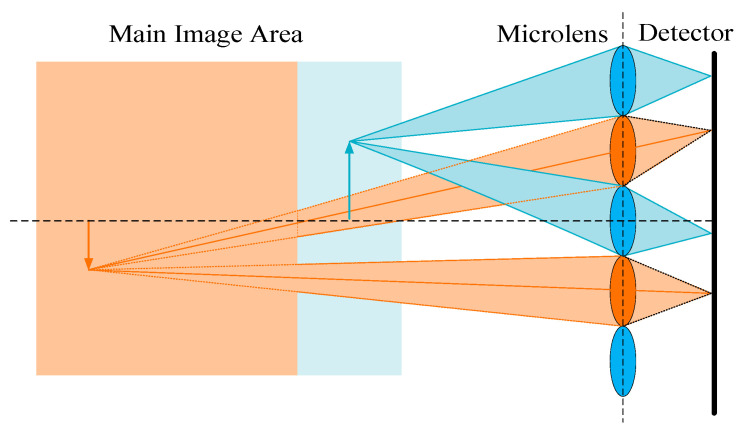
Schematic diagram of microlens with different focal lengths for secondary imaging.

To improve the depth of the system’s field, a multifocal microlens array structure has been developed. The focal lengths of sub-microlens must be set differently in the microlens array so that the depth-of-field boundaries of adjacent lenses can be tied together. With this design, the depth of field of the system is the sum of the depth of field of multiple sub-microlens with different focal lengths, greatly increasing the depth-of-field range. As shown in Figure 8, a microlens array for secondary imaging is composed of two sub-microlens with staggered focal lengths. Secondary images of close targets were acquired using short focal length microlens, while distant targets were acquired using long focal length microlens. The designed focal length value allows the short focal length and long focal length microlens to be connected in the clear imaging range, extending the depth-of-field range.

#### 2.3.3. Design and Optimization of Sub-Microlens

In a light field imaging system, a microlens array plays a major role in determining the imaging quality of the system, and the structure of the individual sub-microlens within the array determines the overall performance. For the sub-microlens design, a standard spherical mirror is used, and the lens type is a plano-convex structure. We calculate the focal length of the sub-microlens using Formula (12) [29]:

(12) $$\frac{1}{f}=(n-1)\left[{\frac{1}{R}}_{1}-\frac{1}{R_{2}}+\frac{(n-1)l}{nR_{1}R_{2}}\right],$$

where *f* represents focal length, *n* represents refractive index, *l* represents thickness, and *R*_1_ and *R*_2_ are the radius of curvature of the surface front and back, respectively. In this case, since the posterior surface is plano-convex, the radius of curvature *R*_2_ is infinite.

**Figure 9:**
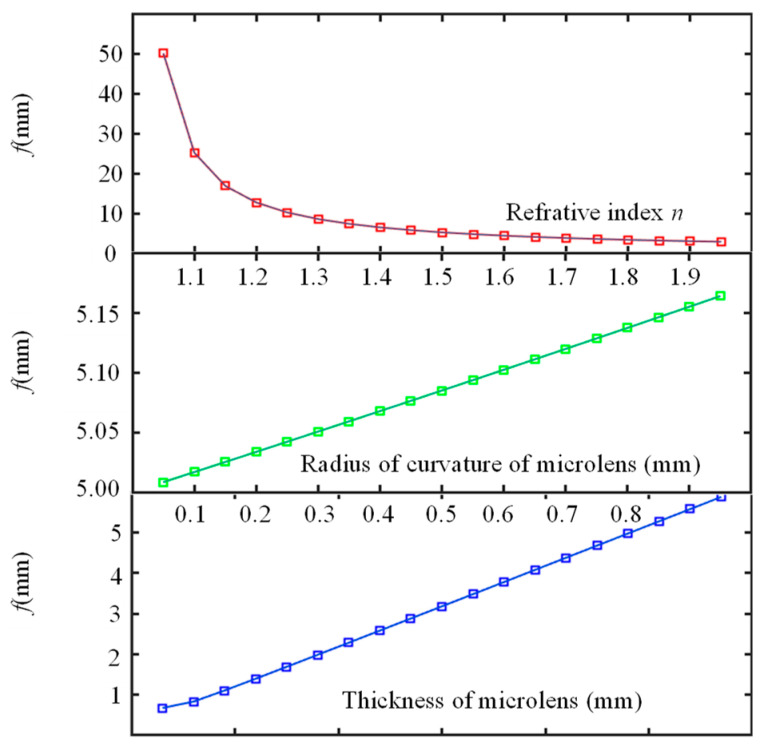
The effect of different parameters on focal length.

Three parameters determine the focal length of the sub-microlens: the refractive index *n*, the thickness *l*, and the radius of curvature *R*. The relationship between focal length and these three parameters can be seen in Figure 9. According to Figure 9, focal length *f* follows linearly from thickness *l* and radius *R* and quadratically from refractive index *n*. When changing *R* to change *f*, increasing the radius of curvature will increase interference between adjacent units, reducing information transmission; when changing *l* to change *f*, due to its thin thickness, sub-microlens cannot be manufactured accurately, resulting in a large error. Therefore, changing *n* is the most appropriate way to adjust the focal length.

The microlens array contains identical sub-microlens, so optimizing the same for each sub-lens can improve the overall image quality. Commonly, microlens arrays are made from PMMA, and their surfaces are typically spherical mirrors. In this case, there is a certain amount of aberration after imaging, which will affect the overall image quality. To further improve imaging quality, the aspheric surface type is considered to optimize the sub-microlens. A surface structure called an even aspheric surface [30] is commonly used for aberration correction and optimization, and its expression is shown in Equation (13).

(13) $$z=\frac{c\cdot r^{2}}{1+\sqrt{1-\left(1+k\right)\cdot c^{2}\cdot r^{2}}}+\sum_{i=1}^{n}a_{2i}r^{2i},$$

where *c* represents the reciprocal of the radius of curvature; *k* represents the surface cone coefficient of the aspheric surface; and $\sum_{i=1}^{n}a_{2i}r^{2i}$ is a multiple term of a surface equation for an aspheric surface.

## 3. Simulation, Analysis, and Discussion

### 3.1. Design and Analysis of Sub-Microlens

**Figure 10:**
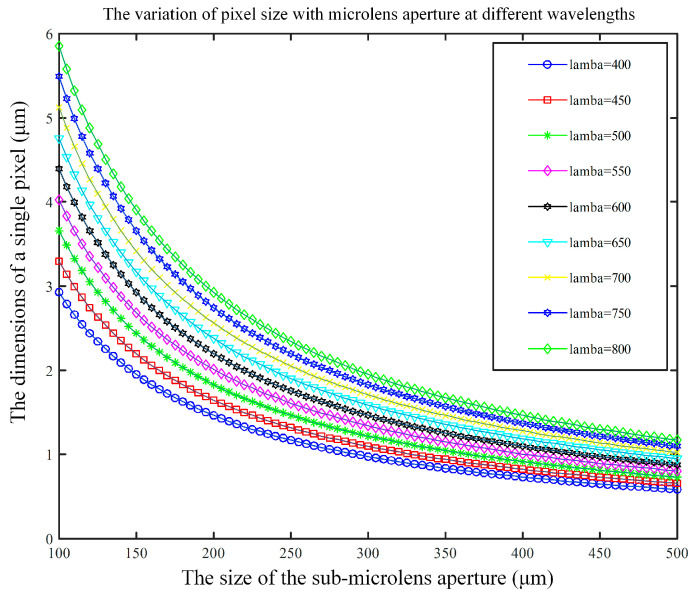
The variation of Airy disk with *d* under different λ.

The aperture *d* of sub-microlens is a very important parameter; the smaller the aperture, the more arrays of microlens there are, and the higher the spatial resolution. Nevertheless, when the aperture of the sub-microlens is small enough, the diffraction phenomenon may affect the imaging. Therefore, the relationship between the aperture and the diffraction phenomenon must be taken into account. During the practical application, the Airy disk is utilized to determine if diffraction influences it. The diffraction phenomenon is not affected when the Airy disk is less than or equal to the size of the image sensor pixel. Light field imaging in this design is performed in the visible light range between 400 nm and 800 nm, with sample intervals of 50 nm. The parameter *B* is set to 0.6 mm. In Figure 10, you can see how the radius of the Airy disk changes with the aperture of the sub-microlens at different wavelengths of incident light. It can be seen that with the increase in the aperture of the sub-microlens, the radius of the Airy disk decreases. An Airy disk’s radius is positively correlated with the incident wavelength; the longer the incident wavelength, the larger the Airy disk’s radius.

This paper uses a detector with pixel sizes of about 5 μm, and we use sub-microlens with an aperture of 100 μm to make the light field imaging system have a higher spatial resolution. Whenever the incident wavelength is between 400 and 700 nm, the radius of the Airy disk equals or is less than the pixel size; thus, diffraction phenomena do not affect imaging.

To study the influence of surface shape on imaging quality, three different surface shapes were designed: standard surface shape, single-sided even aspheric surface, and double-sided even aspheric surface. Each of these three surface structures was simulated using Zemax optical design software, version 21.1, and Table 1 illustrates their parameters.

**Figure 11:**
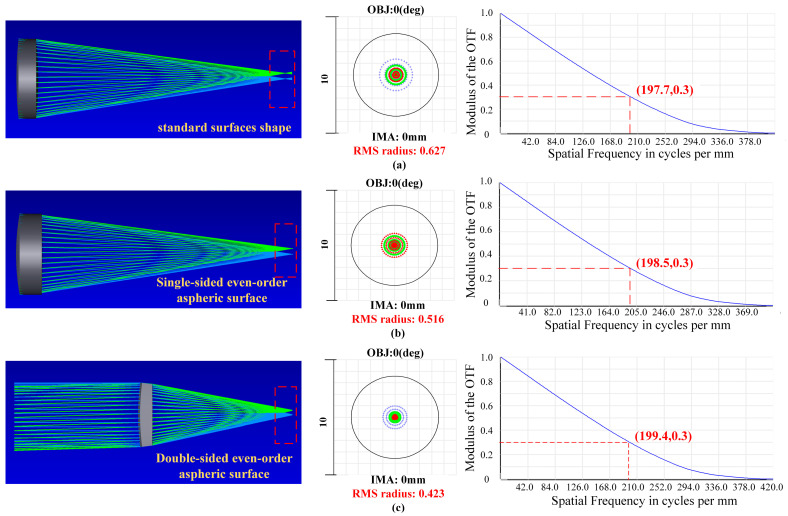
Simulation results of sub-microlens with three different surface shapes. (**a**) MTF curves, spot diagrams, and imaging results for standard surface shape; (**b**) MTF curves, spot diagrams, and imaging results for single-sided even aspheric surfaces; (**c**) MTF curves, spot diagrams, and imaging results for double-sided even aspheric surfaces.

An imaging result of one sub-microlens from a Zemax simulation is shown in Figure 11. According to the results, the light cannot completely focus on the focal point when the ordinary spherical mirror is imaging, which will result in some divergence in the final imaging. Nevertheless, it is effective in solving this problem and improving imaging quality with the use of an aspheric structure, whether it is a single-sided aspheric or a double-sided aspheric structure. For *d* = 100 μm, the RMS radius of a standard spherical surface is 0.627 μm, that of a single-sided even aspheric surface is 0.516 μm, and that of a double-sided even aspheric surface is 0.423 μm. Considering the spot diagrams results, the aspheric surface type has a certain improvement on the imaging quality of the microlens, whereas the double-sided even aspheric effect is superior. When the center wavelength is 550 nm, and the MTF value is greater than 0.3, the eigenfrequency of the standard spherical surface reaches 197.7 lp/mm. The eigenfrequency of single-sided even aspheric surface reaches 198.5 lp/mm. The eigenfrequency of the double-sided even aspheric surface can reach 199.4 lp/mm. Consequently, double-sided even-time aspheric imaging has the best image quality, single-sided even-time aspheric image quality is second, and standard spherical mirror image quality is poor. Combined with the analysis results of the spot diagrams, the double-sided even aspheric structure is selected as the surface shape of the sub-microlens.

Due to processing, assembly and adjustment, and improper handling of the staff, an optical system will exhibit a certain amount of error during the actual production process. Thus, tolerance analysis is an important method of assessing optical system stability. A tolerance assignment table for the double-sided even aspheric surface is shown in Table 2.

**Figure 12:**
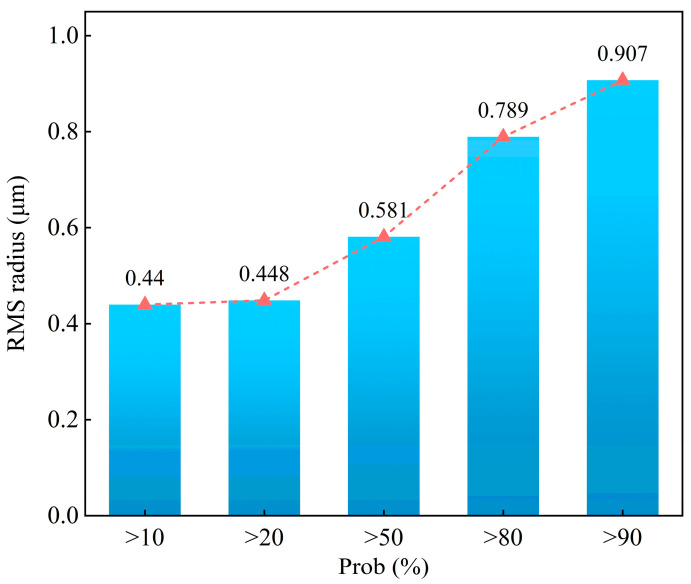
Tolerance analysis results for the double-sided even aspheric surface.

Based on sensitivity and Monte Carlo analysis methods, a tolerance analysis was conducted using the RMS radius of the spot diagrams as a measure of system performance in this study. As shown in Figure 12, the tolerance analysis results are displayed as probabilities for changing the RMS radius of the spot diagrams of the double-sided even aspheric surface. The results indicate that more than 90% of the samples had an RMS radius of less than 0.907 μm, much smaller than the radius of the Airy spot (3.53 μm). There is no problem with the tolerance range as it meets the RMS radius requirements of the spot diagrams for the double-sided even aspheric surface.

**Figure 13:**
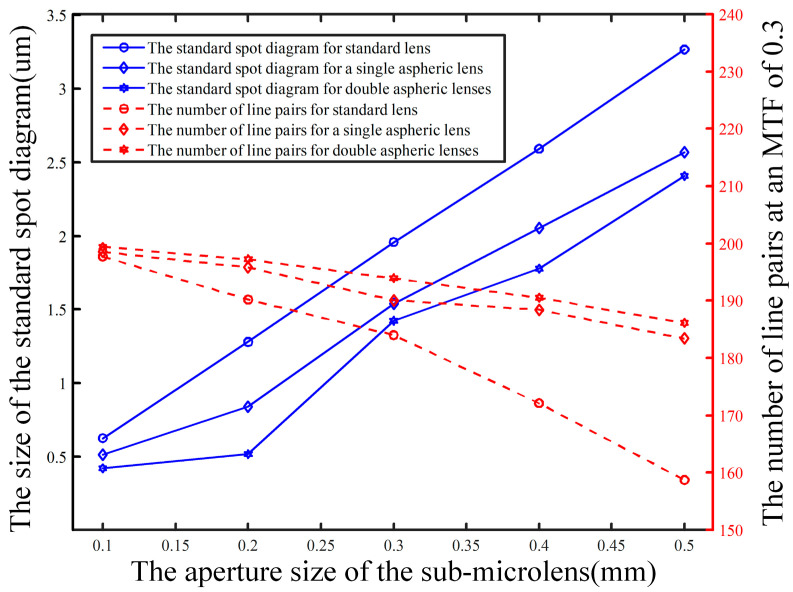
Eigenfrequency and RMS radius of different surface shapes at various apertures.

Further, it should be noted that the aperture of the microlens can also affect the quality of the image. The aperture d was set to 100 μm, 200 μm, 300 μm, 400 μm, and 500 μm, respectively, and the eigenfrequency and RMS radius were calculated for these three different surface shapes. The calculation results and variation law are shown in Figure 13. When the aperture is increased, the eigenfrequency decreases, and the RMS radius increases. In general, the smaller the sub-microlens, the better the imaging quality.

Generally, the smaller the aperture *d* of the sub-microlens, the better the imaging quality and the higher the spatial resolution under the premise of no diffraction. Therefore, we chose a square sub-microlens with an aperture of 100 μm and an even aspheric surface as the unit of the microlens array in this paper.

### 3.2. Design and Analysis of a New Light Field Imaging System

**Figure 14:**
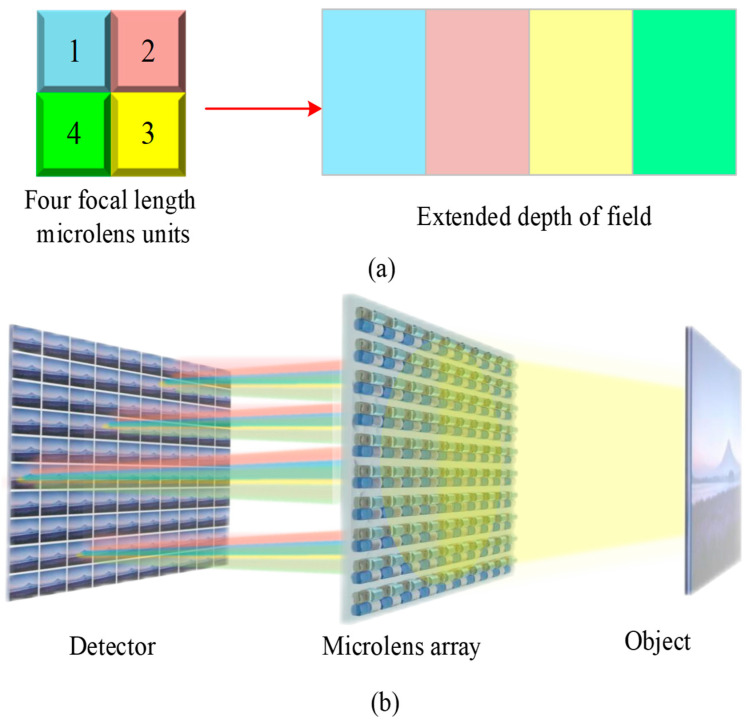
Novel four-focal-length square microlens array structure and imaging optical path. (**a**) four-focal-length sub-lens unit and depth-of-field extension; (**b**) the imaging optical path of the novel light field imaging system.

This paper presents a novel four-focal-length square microlens array structure based on the multifocal length extended depth-of-field theory described in Section 2.3.2. Figure 14 displays the four-focal-length microlens units, the extended depth-of-field range, and the imaging optical path of this structure. The depth-of-field boundaries of the four sub-microlens must be just connected to ensure that the four focal lengths of the square microlens array maximize the depth of field.

**Figure 15:**
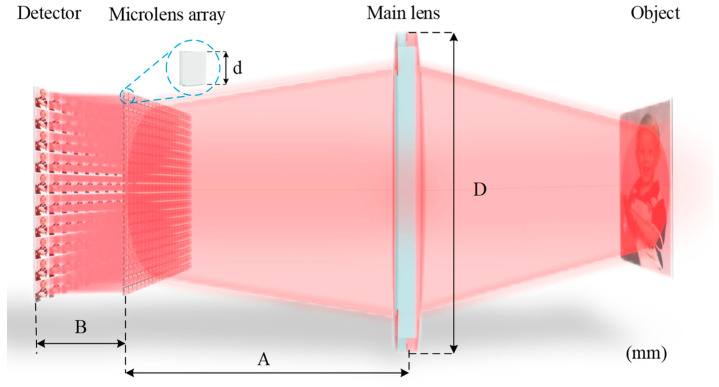
Simulation diagram of the overall structure of the system.

Assuming that the focal lengths of the four sub-microlens in Figure 15 are *f*_1_, *f*_2_, *f*_3_, and *f*_4_, respectively, then they need to meet the following requirements: the left boundary of the depth of field of the second sub-microlens is equal to the right boundary of the depth of field of the first sub-microlens; the left boundary of the depth of field of the third sub-microlens is equal to the right boundary of the depth of field of the second sub-microlens; the left boundary of the depth of field of the fourth sub-microlens is equal to the right boundary of the depth of field of the third sub-microlens, as shown in Equation (14):

(14) $$\left\{\begin{array}{c} a_{0}^{+}f(2)=a_{0}^{-}f(1)\\ a_{0}^{+}f(3)=a_{0}^{-}f(2)\\ a_{0}^{+}f(4)=a_{0}^{-}f(3) \end{array}\right.,$$

where $a_{0}^{+}$() is the left boundary of the depth of field of the sub-microlens and $a_{0}^{-}$() is the right boundary of the depth of field of the sub-microlens.

In Section 3.1 above, the analysis results indicate that the single focal length microlens array has sub-microlens with *f* = 0.5 mm and *d* = 100 μm. As calculated by Equation (14), *f*_2_ = 0.46 mm, *f*_3_ = 0.42 mm, and *f*_4_ = 0.54 mm. In Table 3, we compare the depth-of-field results of single focal length and four-focal-length microlens array. Calculations indicate that the depth of field of the four-focal-length microlens array is 6.57 times greater than that of the single microlens array. Compared to a single focal length microlens array, a four-focal-length microlens array significantly improves the light field imaging system depth of field.

Using Zemax, the novel light field imaging system was simulated and validated after parametric analysis and system design. This simulation is intended to demonstrate the impact of the structure of the light field imaging system on the imaging effect, so a single focal length microlens array is used. An overview of the simulation parameters for this system can be found in Table 4. An ideal lens makes up the main lens, while a PMMA square and standard lens serve as the sub-microlens.

By using Zemax version 21.1’s sequential mode simulation after setting the system parameters, the overall simulation optical path can be seen in Figure 15.

**Figure 16:**
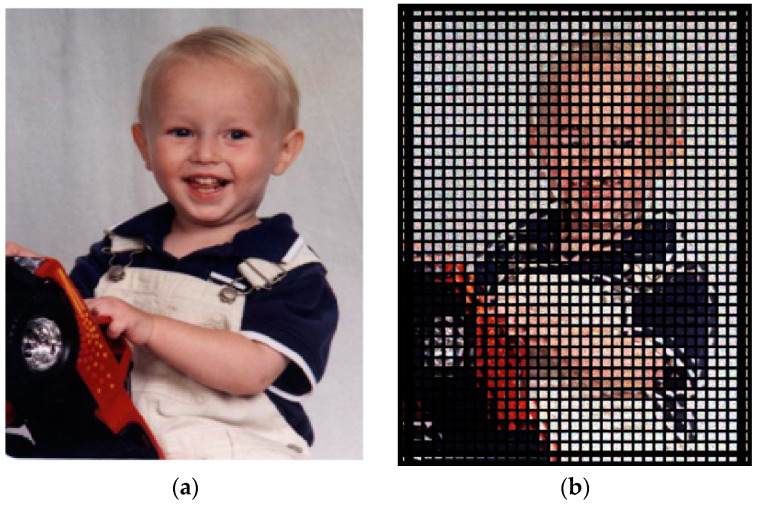
The results of the system simulation. (**a**) The original image; (**b**) the light field image after simulation.

Using geometric bitmap analysis in Zemax, simulation imaging was performed, and its imaging quality was evaluated. Figure 16a shows an image with geometric bitmap analysis used for image simulation. The original image is placed on the detector plane of the novel light field imaging system, and the image resolution setting is the same as that of the image sensor. An image of the light field obtained after the simulation can be seen in Figure 16b. This paper demonstrates that a light field imaging system can be designed to obtain the light field information of an image and provide a clear image.

**Figure 17:**
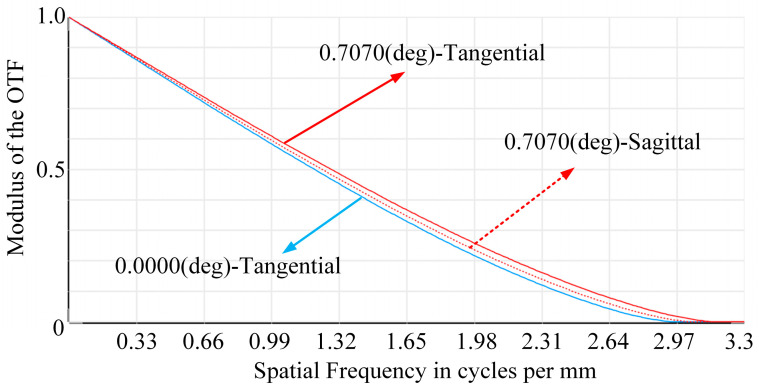
MTF curves for a single sub-microlens.

To further assess the imaging quality of the light field imaging system, the MTF curve was used to evaluate imaging quality. Figure 17 shows the MTF curve for the sub-microlens in three visible fields of view. When a single sub-microlens’ MTF value is greater than 0.3, its eigenfrequency can reach more than 1.7 lp/mm, indicating high image quality.

**Figure 18:**
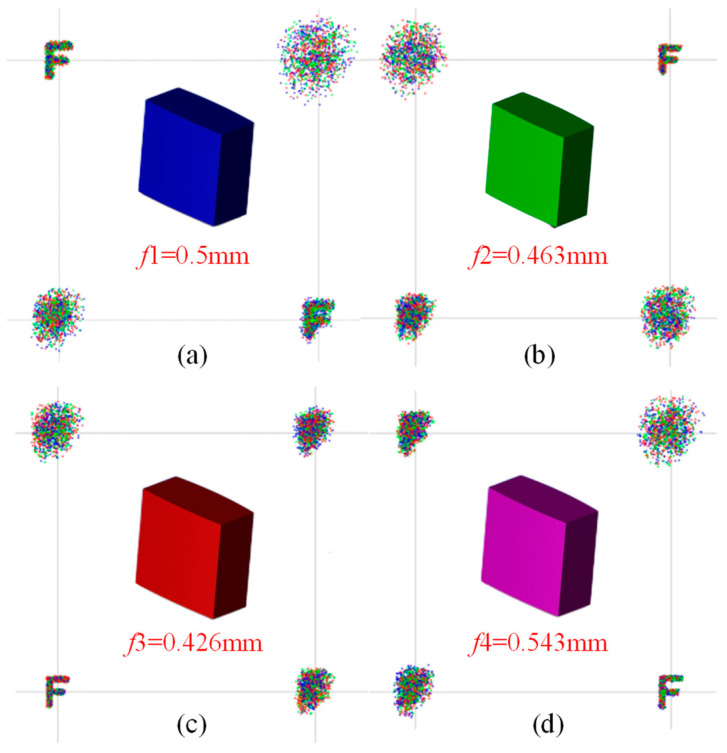
Geometric bitmap image simulation results. (**a**) Focus on sub-microlens 1; (**b**) focus on sub-microlens 2; (**c**) focus on sub-microlens 3; (**d**) focus on sub-microlens 4.

Additionally, the non-sequential mode of Zemax was employed to construct a novel light field imaging system with a four-focal-length square microlens array, and its imaging effect was assessed. As described in Section 3.2, four sub-microlens with different focal lengths are designed, and the imaging results after simulation are shown in Figure 18.

Figure 18 shows that clear imaging of different targets can be achieved by focusing the image on different sub-microlens. In subsequent image processing, other types of image information can be obtained by extracting images at different positions for reconstruction to achieve clear imaging of targets in various depths of the field. By combining a microlens array structure with a light field imaging system, this paper can achieve high resolution, wide depth of field, and high imaging quality.

Although the simulation results demonstrate the superiority of the new microlens array structure and the light field imaging system, there are still processing limitations, such as high costs and complexity. As advanced optical fabrication technology develops, the structure proposed in this paper will become increasingly important for light field imaging.

## 4. Conclusions

This paper presented a novel four-focal-length square microlens array and light field imaging system to overcome the limitations of traditional imaging systems in terms of low resolution, small depth of field, and poor image quality. Various components make up the system, including the main lens, a novel microlens array, and a detector. With its focused light field structure, the system is capable of high-resolution imaging, and the new microlens array achieves high-quality imaging with a large depth of field. This system was simple, compact, and had a high spatial resolution and image quality, which made it superior to similar systems and kept up with the future development trends for light field imaging. A novel microlens array and imaging system for light fields were developed using Zemax and geometric bitmap simulation. A micro square array structure designed in this paper achieves more than 90% pixel utilization. Compared to using a single focal length, a four-focal-length microlens array yields 6.57 times more depth of field. With its high performance and ease of construction, this system has extensive applications in industrial inspections.

## Figures and Tables

**Table 1:** Parameters for three different types of sub-microlens.

	Standard Surface Shape	Single-Sided Even Aspheric Surfaces	Double-Sided Even Aspheric Surfaces
Diameter (μm)	100	100	100
Thickness (mm)	0.05	0.05	0.05
Material	PMMA	PMMA	PMMA
Radius of curvature	Front surface: 0.548 Rear surface: ∞	Front surface: 0.258 Rear surface: ∞	Front surface: 0.511 Rear surface: −0.505
Cone coefficient	—	Front surface: −1 Rear surface: 0	Front surface: −2 Rear surface: −2

**Table 2:** Tolerance assignment table for the double-sided even aspheric surface.

Parameter	Specification
Radius of the surface (mm)	±0.002
Conic of the surface	±0.05
Thickness (mm)	±0.005
Index	±0.001

**Table 3:** Comparison of the depth of field of two microlens arrays.

	$\mathrm{DOF}a_{0}^{-}$ (mm)	$\mathrm{DOF}a_{0}^{+}$ (mm)	$\mathrm{DOF}a_{0}$
Single focal length	2.420	3.948	1.528
Four focal length	0.523	10.753	10.05

**Table 4:** Parameters of the novel light field imaging system.

Component	Parameter	Value
Main lens	Focal distance (mm)	100
Microlens array	Aperture (mm)	20
	Focal length (mm)	0.5
	Diameter (mm)	0.1
	Number of sub-microlens	100 × 100
Imaging sensor	Spatial resolution	1280 × 1024
	Image size (μm)	4.8

## Data Availability

The original contributions presented in the study are included in the article, further inquiries can be directed to the corresponding author.
